# Interactions between *Lactobacillus crispatus* and Bacterial Vaginosis (BV)-Associated Bacterial Species in Initial Attachment and Biofilm Formation

**DOI:** 10.3390/ijms140612004

**Published:** 2013-06-05

**Authors:** António Machado, Kimberly Kay Jefferson, Nuno Cerca

**Affiliations:** 1 Centre of Biological Engineering, IBB—Institute for Biotechnology and Bioengineering, University of Minho, Campus de Gualtar 4710-057, Braga, Portugal; E-Mail: machadobq@deb.uminho.pt; 2 Department of Microbiology and Immunology, Virginia Commonwealth University, Richmond, VA 23298-0678, USA; E-Mail: kkjefferson@vcu.edu

**Keywords:** *Lactobacillus* spp., *Gardnerella vaginalis*, BV anaerobes, initial adhesion, epithelial cell line, fluorescence *in situ* hybridization, peptide nucleic acid, quantitative-PCR

## Abstract

Certain anaerobic bacterial species tend to predominate the vaginal flora during bacterial vaginosis (BV), with *Gardnerella vaginalis* being the most common. However, the exact role of *G. vaginalis* in BV has not yet been determined. The main goal of this study was to test the hypothesis that *G. vaginalis* is an early colonizer, paving the way for intermediate (e.g., *Fusobacterium nucleatum*) and late colonizers (e.g., *Prevotella bivia*). Theoretically, in order to function as an early colonizer, species would need to be able to adhere to vaginal epithelium, even in the presence of vaginal lactobacilli. Therefore, we quantified adherence of *G. vaginalis* and other BV-associated bacteria to an inert surface pre-coated with *Lactobacillus crispatus* using a new Peptide Nucleic Acid (PNA) Fluorescence *In Situ* Hybridization (FISH) methodology. We found that *G. vaginalis* had the greatest capacity to adhere in the presence of *L. crispatus*. Theoretically, an early colonizer would contribute to the adherence and/or growth of additional species, so we next quantified the effect of *G. vaginalis* biofilms on the adherence and growth of other BV-associated species by quantitative Polymerase Chain Reaction (qPCR) technique. Interestingly, *G. vaginalis* derived a growth benefit from the addition of a second species, regardless of the species. Conversely, *G. vaginalis* biofilms enhanced the growth of *P. bivia*, and to a minor extent of *F. nucleatum.* These results contribute to our understanding of BV biofilm formation and the progression of the disorder.

## 1. Introduction

Bacterial vaginosis (BV) is the most common vaginal disorder in women of reproductive age but its etiology is still unclear [1]. However, BV is characterized by a decrease in beneficial vaginal bacteria, such as *Lactobacillus cripatus*, and by an increase of the number of anaerobic bacteria, such as *Gardnerella vaginalis*, *Mobiluncus mulieris*, *Atopobium vaginae*, *Prevotella bivia* and *Fusobacteria nucleatum* [2–4]. BV is typically a polymicrobial condition [5,6]. Recently it has been found that multi-species microbial biofilms are involved in BV [4]; however, the process by which this multi-species biofilm is established remains unknown. In general, single-species biofilm formation involves two main independent steps: initial adhesion to the surface and biofilm accumulation [7]. In contrast, multi-species biofilm formation may be more complex and depend upon interactions between the species involved. The most thoroughly studied clinically relevant polymicrobial biofilm is the oral biofilm associated with periodontitis [8]. During the development of these biofilms, early colonizers first adhere to the tooth pellicle and provide a surface to which intermediate colonizers can adhere as well as producing more optimal conditions for growth of successive species [9,10]. This community in turn provides an environment conducive to the adherence and growth of secondary colonizers. Similar to oral biofilms, it has been hypothesized that *G. vaginalis* is the initial colonizing species and that *G. vaginalis* biofilms are conducive to growth, adherence and/or biofilm formation by other BV anaerobes, but this has yet to be demonstrated [4].

The main goal of our work was to assess the potential of bacterial species commonly found in BV as early or late colonizers. We first quantified the initial adhesion potential to an inert surface pre-coated with *Lactobacillus crispatus* and then compared single-species or dual-species biofilms formation in order to assess the potential symbiotic interactions between BV-associated bacterial species.

## 2. Results and Discussion

### 2.1. Determination of Early Adhesion Potential to Surface Coated with *L. crispatus*

In 1983, Spiegel and colleagues postulated that bacterial vaginosis was a polymicrobial infection, where *G. vaginalis* was the prevalent species [11]. However, the etiology of BV remains unknown, and it is unclear which, if any of the BV-associated anaerobes are capable of disrupting an established *Lactobacillus* population and initiate colonization on the vaginal epithelium. Several species of lactobacilli may colonize the healthy vagina, however each species differs in its probiotic activity due to differences in their abilities to endure changes in conditions, such as pH variations due to menstruation or sexual intercourse, and due to differences in their abilities to produce antimicrobial compounds such as lactic acid, hydrogen peroxide and bacteriocins [12]. *L. crispatus* is able to produce several antimicrobial compounds and is inversely associated with BV [13]. We therefore chose this species as a representative for use in our study. We attempted to evaluate the early adhesion to an inert surface pre-coated with *L. crispatus* by know BV associated anaerobes at different concentrations (1 × 10^3^ and 3 × 10^9^ CFU/mL). As shown in Table 1, *G. vaginalis* was more adherent at either concentration than the other BV anaerobes (*ANOVA Tukey* statistical test, *p* < 0.05), followed by *F. nucleatum* and *P. bivia*, respectively. These results are in agreement with several previous studies [14–16] supporting evidence that *G. vaginalis* has significant initial adhesion potential. This suggests that *G. vaginalis* could be the early colonizer in the progression of BV. Although *A. vaginae* and *M. mulieris* are often associated with BV [17–19], their capacity to adhere to glass pre-coated with *L. crispatus* was the lowest of all tested anaerobes, suggesting that they are not strong candidates as early colonizers in BV. Interestingly, *M. mulieris* appeared to displace *L. crispatus* more effectively than any of the other anaerobes tested, including *G. vaginalis* (*ANOVA Tukey* statistical test value, *p* < 0.05; see Table 2). Since this species did not adhere as well, this suggests that it may secrete some soluble factors that displace the lactobacilli. However, these *in vitro* experiments are limited in that the bacteria were allowed to adhere to glass rather than vaginal epithelium and adherence to vaginal epithelium is likely influenced by a number of host-related and bacteria-specific factors, such as mucus production and the involvement of specific receptors on the epithelial surface [1,3].

### 2.2. *G. vaginalis* Mediated Dual Species Biofilms

Studies have shown the prevalence of biofilm formation in BV samples, exposing *G. vaginalis* as a main component of these biofilms, leading to the hypothesis that *G. vaginalis* initiates the biofilm allowing successive species to adhere and proliferate [4,20]. However, this has yet to be determined experimentally. We examined whether synergistic or antagonistic interactions would contribute to or prevent growth of BV anaerobes within an early-stage *G. vaginalis* biofilm. *G. vaginalis* biofilms were allowed to develop for 24 h, after which a second anaerobe was introduced and co-cultured in the system for an additional 24 h. Quantitative PCR analysis was used to determine the number of *G. vaginalis* and the second species within the biofilm. Notably, *G. vaginalis* growth was augmented by the incorporation of a second anaerobe after the initial 24 h biofilm formation (Table 3). *G. vaginalis* growth increased in the presence of every species (≈3 fold increase) but the greatest increase was found in the presence of *P. bivia* (3.83-fold increase) and *M. mulieris* (3.78-fold increase) as shown in Table 3. Interestingly, *F. nucleatum* and *P. bivia* reached higher numbers when co-cultured with *G. vaginalis* strains, showing ≈2 and ≈4 fold increases (see Table 3), respectively. This is in agreement with a report from Pybus and Onderdonk revealing a symbiotic relationship between *G. vaginalis* and *P. bivia* [17] and suggesting that symbiotic relationships established between *G. vaginalis* and other anaerobes in BV biofilms could contribute to the progression of BV. The results with *F. nucleatum* were interesting as well. Although *F. nucleatum* has not been extensively studied in BV infection, it plays a key role in the establishment of oral biofilms as a bridging species [21]. In fact, Foster and Kolenbrander [21] demonstrated that *F. nucleatum* is capable of co-aggregating with pathogenic bacteria and becoming a dominant member of the oral multispecies biofilm after several days of incubation although it commonly failed to grow by itself in biofilms. Similarly, our results suggest that *F. nucleatum* could be capable of joining an initial biofilm and eventually establishing a symbiotic relationship with *G. vaginalis*. Again, our study is limited in its complexity and lacks host-specific factors, but it does suggest that certain BV-related species can cooperate and this may provide some insight regarding the ability of these bacterial species to become dominant in an environment normally dominated by lactobacilli.

## 3. Experimental Section

### 3.1. Culture of Bacterial Strains

*L. crispatus* EX533959VC06 was grown in Man, Rogosa and Sharpe both (MRS; Sigma-Aldrich; Buchs, Switzerland) at 37 °C under anaerobic conditions (AnaeroGen Atmosphere Generation system; Oxoid; Cambridge, UK) for 24–48 h prior to adhesion assays. Also, *G. vaginalis* 101, *Atopobium vaginae* FA, *Mobiluncus mulieris* ATCC 26-9, *Prevotella bivia* ATCC 29303 and *Fusobacteria nucleatum* 718BVC were grown in supplement Brain Heart Infusion (sBHI; Oxoid) and incubated at 37 °C under anaerobic conditions (AnaeroGen Atmosphere Generation system; Oxoid) for 24–48 h prior to adhesion assays. Prior to displacement/blockage assays, all strains were harvested by centrifugation (4000*g*, 12 min, at room temperature), washed twice with sterile PBS. The pellet from each bacteria culture was resuspended in PBS and its concentration was adjusted to 1 × 10^9^ CFU/mL by optical density at 600 nm using a microplate reader (Tecan; Zurich, Switzerland).

### 3.2. Early Adhesion Assays

Aliquots of 40 μL of *L. crispatus* culture media with a concentration of 1 × 10^9^ CFU/mL were added to each well from the 8 chamber glass slide intended to the adhesion assay. Then, 8 chamber glass slides were incubated for 4h at 37 °C, in anaerobic conditions, and 120 rpm. Non-adherent lactobacilli were removed by washing with 400 μL of sterile PBS and subsequently a second adhesion step was performed, using one BV-associated anaerobe with two different concentrations (1 × 10^3^ or 1 × 10^9^ CFU/mL), for 30 min at 37 °C, in anaerobic conditions and 120 rpm at the same range of concentrations. Finally, each well of the incubated 8 chamber slide was carefully washed twice with 40 μL of sterile PBS to remove non-adherent bacteria and let to air-dry before FISH hybridization procedure. Adhesion controls were performed simultaneously in each 8 chamber slide adding each bacterium individually and maintaining the same experimental conditions. All these assays were elaborated with duplicates and each assay was repeated three independent times.

### 3.3. Fluorescent *in Situ* Hybridization and Adhered Bacteria Quantification

The 8 chamber glass slides containing the adhered bacteria were first fixed and hybridized with Lac663 and Gard162 PNA probes, that we previously developed and optimized [22]. Briefly, the adhered bacteria glass slides were fixed with 4% paraformaldehyde followed by 50% methanol, for 10 min, at room temperature, on each solution. After the fixation step, the glass slides were covered with 20 μL of hybridization solution with PNA probe (200 nM). Hybridization was performed at 60 °C for 90 min and for washing (60 °C for 30 min) and a fresh solution was prepared less than 24 h before use. Finally, the glass slides were allowed to air dry in the dark. An additional DAPI staining step was done at the end of the hybridization procedure, covering each glass slide with 20 μL of DAPI (2.5 μg/mL, Sigma) for 5 min at room temperature in the dark, followed by five washing steps with 20 μL of PBS. Then immediate observation was elaborated in the fluorescence microscope. Microscopic visualization was performed using an EVOS*fl* fluorescence microscope (AMG; Bothell, WA, USA) equipped with a CCD camera (Sony ICX285AQ color; Fujian, China) and filters capable of detecting the two PNA probes and DAPI staining. All these assays were repeated three times, on separate days, with three fields of view assessed each time. In each experimental assay, a negative control was performed simultaneously with each step previous described, but where no probe or DAPI staining were added in the hybridization step. Bacteria adhered cells quantification was realized through the National Institutes of Health image analysis software ImageJ (version 1.451) [23].

### 3.4. *G. vaginalis* Mixed Species Biofilms Assays and Quantitative-PCR Procedure

The formation of *G. vaginalis* mixed biofilms were performed in a chemically medium (CDM), previously developed by Geshnizgani and Onderdonk [24]. An initial inoculation of 100 μL overnight of *G. vaginalis* 101 growth was placed into 10 mL of CDM. Then, 2 mL of *G. vaginalis* strain were put in each well of 6-well plate and incubated for 24 h, at 37 °C, in anaerobic conditions. After 24 h, CDM media was changed in each well plate by a fresh CDM media and an inoculation of 50 μL overnight culture from a different second anaerobe was performed. Next, the 6-well plates were incubated for another 24 h, at 37 °C, in anaerobic conditions. Finally, CDM media and planktonic cells were removed from all 6-well plates and then DNA was extracted from biofilm samples by using a Dneasy blood and tissue kit (Qiagen; Hilden, Germany), following manufacturer instructions. All qPCR assays were performed using a Taq 2× Master Mix (BioLabs; Ipswich, MA, USA) on an iCycler iQ5 real-time detection system (Bio-Rad; Hercules, CA, USA). Each 25 μL reaction mixture contained 12.5 μL Taq 2× Master Mix, 1.0 μL of 10 μM from forward and reverse primers (see Table 4), 2 μL template DNA, 8.5 μL of nuclease-free water. Temperature cycling for all assays was 95 °C for 10 min, followed by 40 cycles at 95 °C for 15 s, 54 °C for 30 s and 72 °C for 15 s. Negative controls (no template DNA) were run with every assay to check for contamination. Assay results were expressed as threshold cycle number (*C*_t_) of the 16S rRNA gene copies amplification per template DNA sample. All these assays were elaborated with duplicates and each assay was repeated three independent times.

### 3.5. Statistical Analysis

The data was analysed using a two-tailed ANOVA or Student’s *t*-test with SPSS statistical software (version 17.0) and expressed as mean ± standard deviation (SD). *p* < 0.05 was considered significant.

## 4. Conclusions

Our results suggest that *G. vaginalis* may be more suited as an early colonizer relative to the BV-associated anaerobes tested in the initial adhesion assay, and that it may play a key role in the early establishment of BV biofilms, as previously postulated by Swidsinski *et al.* [4]. All anaerobes tested enhanced biofilm formation by *G. vaginalis* and also *G. vaginalis* biofilms enhanced the growth of *P. bivia* and to a minor extent of *F. nucleatum.* These observations provide some clarification regarding the ability of each individual BV-associated anaerobe tested to adhere in the presence of a protective layer of lactobacilli and regarding the ability of *G. vaginalis* biofilms to thrive in presence of other anaerobes.

## Figures and Tables

**Table 1:** Blockage of adherence of bacterial vaginosis (BV)-associated anaerobes to glass by adherent *L. crispatus*. The number of each BV-associated anaerobes that adhered per cm^2^ of glass (±standard deviation) is shown on the left and the percentage of bacteria that adhered when the glass was pre-coated with *L. crispatus* relative to the control (±standard deviation) is shown on the right.

	Number of BV anaerobe per cm^2^	Percentage adherent to *L. crispatus*-coated glass
**High inocula**		
*G. vaginalis* 101	5.71 × 10^7^ (±2.14 × 10^4^)	86.86% c,d,e,f (±14.14)
*A. vaginae* FA	6.85 × 10^6^ (±3.38 × 10^5^)	48.74% a,b (±3.36)
*M. mulieris* ATCC 26-9	5.76 × 10^6^ (±1.21 × 10^5^)	82.22% a,b (±0.37)
*P. bivia* ATCC 29303	1.64 × 10^7^ (±6.29 × 10^5^)	101.67% b (±28.19)
*F. nucleatum* 718BVC	2.54 × 10^7^ (±9.41 × 10^5^)	68.83% a,b (±5.60)
**Low inocula**		
*G. vaginalis* 101	6.89 × 10^6^ (±1.26 × 10^6^)	72.33% (±4.36)
*A. vaginae* FA	1.47 × 10^5^ (±9.65 × 10^4^)	50.27% a (±3.97)
*M. mulieris* ATCC 26-9	1.33 × 10^6^ (±5.05 × 10^4^)	70.15% (±7.80)
*P. bivia* ATCC 29303	2.99 × 10^6^ (±1.44 × 10^5^)	84.17% (±1.57)
*F. nucleatum* 718BVC	2.68 × 10^6^ (±5.52 × 10^4^)	60.15% a (±0.28)

High inocula = 1 × 10^9^ CFU/mL, Low inocula = 1 × 10^3^ CFU/mL.

a *p* < 0.05 when using *t*-student statistical analysis (95% confidence interval) for comparison of control and bacteria tested in the adhesion assay;

b *p* < 0.05 analysed using ANOVA Tukey statistical test (95% confidence interval) for comparison with *G. vaginalis* strain tested in the adhesion assay;

c *p* < 0.05 analysed using ANOVA Tukey statistical test (95% confidence interval) for comparison with *A. vaginae* strain tested in the adhesion assay;

d *p* < 0.05 analysed using ANOVA Tukey statistical test (95% confidence interval) for comparison with *M. mulieris* strain tested in the adhesion assay;

e *p* < 0.05 analysed using ANOVA Tukey statistical test (95% confidence interval) for comparison with *P. bivia* strain tested in the adhesion assay;

f *p* < 0.05 analysed using ANOVA Tukey statistical test (95% confidence interval) for comparison with *F. nucleatum* strain tested in the adhesion assay.

**Table 2:** Displacement of adherent *L. crispatus* by BV-associated anaerobes. Following the addition of a BV-associated anaerobe, the number of remaining *L. crispatus* was counted and compared to the *L. crispatus* control counting (7.36 × 10^7^ ± 9.97 × 10^4^). The percentage (±standard deviation) of *L. crispatus* that remained adherent after addition of each BV anaerobe at high or low inocula is shown below.

	Percentage of *L. crispatus* remaining after addition of BV anaerobe
**High inocula**	
*G. vaginalis* 101	88.60% b,c (±5.14)
*A. vaginae* FA	99.29% a (±7.26)
*M. mulieris* ATCC	26-9 76.62% a (±11.93)
*P. bivia* ATCC 29303	94.86% (±20.60)
*F. nucleatum* 718BVC	97.65% (±7.41)
**Low inocula**	
*G. vaginalis* 101	101.51% b,c (±28.52)
*A. vaginae* FA	71.18% a (±12.54)
*M. mulieris* ATCC	26-9 68.48% a (±12.79)
*P. bivia* ATCC	29303 97.39% (±2.44)
*F. nucleatum* 718BVC	98.34% (±9.52)

High inocula = 1 × 10^9^ CFU/mL, Low inocula = 1 × 10^3^ CFU/mL.

a *p* < 0.05 analysed using ANOVA Tukey statistical test (95% confidence interval) for comparison with *G. vaginalis* strain tested in the adhesion assay;

b *p* < 0.05 analysed using ANOVA Tukey statistical test (95% confidence interval) for comparison with *A. vaginae* strain tested in the adhesion assay;

c *p* < 0.05 analysed using ANOVA Tukey statistical test (95% confidence interval) for comparison with *M. mulieris* strain tested in the adhesion assay.

**Table 3:** Results of the quantitative PCR (qPCR) from mixed biofilm formation assays with *Gardnerella vaginalis* 101 and a second BV anaerobe. All experiments were done in triplicate.

Biofilm	Single specie biofilm		Multispecies biofilm		% GV in mixed biofilm
	
	GV control CT	2nd anaerobe control CT	GV fold increase	2nd anaerobe fold increase	
*G. vaginalis* (48 h) & *M. mulieris* (24 h)	14.13 (±0.12)	31.99 (±1.09)	3.78 (±1.10) a	0.89 (±0.17)	99.9997

*G. vaginalis* (48 h) & *A. vaginae* (24 h)	14.13 (±0.12)	26.38 (±0.33)	3.38 (±0.79) a	1.37 (±0.17)	99.9844

*G. vaginalis* (48 h) & *P. bivia* (24 h)	14.13 (±0.12)	24.84 (±0.03)	3.82 (±0.03) a	4.20 (±0.92) a	99.8960

*G. vaginalis* (48 h) & *F. nucleatum* (24 h)	14.13 (±0.12)	24.24 (±2.57)	3.39 (±0.28) a	1.63 (±0.44)	99.9236

Legend—GV, *G. vaginalis* 101; CT, threshold cycle; (±standard deviation), standard deviation from the average values from triplicate assays are in parenthesis after the average value.

a *p* < 0.05 when using *t*-student statistical analysis (95% confidence interval) for comparison of control and bacteria tested in the biofilm assay.

**Table 4:** Set of primers used in this study according to the Ribosomal Database Project II (RDPII) for quantitative real-time PCR.

Bacteria target	qPCR primers	DNA target	Accession number in RDPII	Localization in RDPII sequence
*G. vaginalis*	Fw 5′-CACATTGGGACTGAGATACGG-3′	16S rRNA	S002289761	325–345
*G. vaginalis*	Rv 5′-AGGTACACTCACCCGAAAGC-3′	16S rRNA	S002289761	470–490
*M. mulieris*	Fw 5′-CGTGCTTAACACATGCAAGTCG-3′	16S rRNA	S000110434	44–65
*M. mulieris*	Rv 5′-GCTGGCTTTCACGACAGACG-3′	16S rRNA	S000110434	1073–1091
*A. vaginae*	Fw 5′-TATATCGCATGATGTATATGGG-3′	16S rRNA	S000607439	184–205
*A. vaginae*	Rv 5′-CATTTCACCGCTACACTTGG-3′	16S rRNA	S000607439	658–677
*P. bivia*	Fw 5′-CGCACAGTAAACGATGGATG-3′	16S rRNA	S000414458	806–825
*P. bivia*	Rv 5′-ATGCAGCACCTTCACAGATG-3′	16S rRNA	S000414458	1032–1051
*F. nucleatum*	Fw 5′-ATTTGTAGGAATGCCGATGG-3′	16S rRNA	S001577261	694–713
*F. nucleatum*	Rv 5′-TACTTATCGCGTTTGCTTGG-3′	16S rRNA	S001577261	842–861

Searched through RDPII (last accession, December 2012) with the following data set options: Strain—Both; Source—Both; Size—> 1200bp; Quality—Both.
